# Longitudinal 2-Year Follow-up on the Effect of a Non-Randomised School-Based Physical Activity Intervention on Reducing Overweight and Obesity of Czech Children Aged 10–12 Years

**DOI:** 10.3390/ijerph10083667

**Published:** 2013-08-16

**Authors:** Erik Sigmund, Dagmar Sigmundová

**Affiliations:** Center for Kinanthropology Research, Institute of Active Lifestyle, Faculty of Physical Culture, Palacky University in Olomouc, Tr. Miru 115, Olomouc 77111, Czech Republic; E-Mail: dagmar.sigmundova@upol.cz

**Keywords:** obesity, physical activity, after-school care, steps, Yamax pedometer

## Abstract

*Background:* This study assessed whether the benefits of a 2-year longitudinal non-randomised school-based physical activity (PA) intervention programme to reduce overweight and obesity were still apparent two years after completion of the controlled intervention. *Methods:* The study involved 84 girls (G) and 92 boys (B) aged 10–12 years who had participated in the PA intervention in 2006–2008 as 6- to 9-year olds and were included in the intervention (I) (43 G and 45 B) and the control (C) groups (41 G and 47 B). Participants’ overweight/obesity was assessed using the percentile graph of Body Mass Index (BMI) from the World Health Organization for girls and boys aged 5–19. Logistic regression (Enter method) determined the overweight/obesity occurrence in a follow-up measurement (2010) two years after completion of the controlled intervention was used. *Results:* Two years after the controlled PA intervention had finished, the intervention children were less likely to be overweight/obese than the control children (2.3%_GI_ *vs.* 17.1%_GC_, 6.7%_BI_ *vs.* 23.4%_BC_, odds ratio: 0.25; 95% confidence interval: 0.12; 0.53; *p* < 0.001). *Conclusions:* The current study indicates favourable effects of an everyday school-based PA intervention programme on lower overweight/obesity incidence, which was maintained two years after the end of the direct involvement of the researchers.

## 1. Introduction

Although the worldwide prevalence of childhood obesity has considerably increased during the past three decades [1,2,3,4], along with objectively measured trends of a decrease in their physical activity (PA) [4,5], effective obesity-reducing public health interventions and strategies are still limited. Specific causes of obesity are, at a population level, consistent with a long-term positive energy balance that could be a result of a low level of daily PA and prevailing sedentary behaviour [6,7]. Therefore, effective obesity-reduction interventions in children may need to incorporate moderate-to-vigorous PA [7] to permanently increase the level of daily PA [8]. Such findings provide reasons to continue the search for effective strategies and evaluated programmes that could increase PA [7,8,9] and concomitantly reduce the incidence of childhood obesity [8,10,11].

Schools provide optimum tools and settings for the design, implementation, and evaluation of public health interventions to all 6- to 11-year-old children, independent of the child’s race, gender, and socio-economic status due to the long compulsory school attendance [9,11]. Children aged 6 to 11 years (before the onset of puberty) are in a suitable age for the performance of school-based PA (*i.e.*, physical education lessons, school sports competitions) and leisure-time PA as these interventions have a positive effect on higher leisure-time PA in adulthood [9,12,13]. In the long term, throughout the many years of compulsory school attendance, the school environment can aid to support educational activities and practical implementation of nutritional, PA and social interventions [9,11,14]. Indeed, multi-component school-based PA, nutrition and healthy behaviour intervention programmes appear to be more effective in the reduction of obesity levels of preadolescent children when compared with similar family-based or community-based programmes [9,10,11,15,16]. However, to demonstrate any sustained effects of such longitudinal school-based interventions on obesity reduction, repeated follow-up measurements are essential after the intervention has been completed [10,11,17]. Such repeated follow-ups that could trace and gauge, longitudinally, the impact (or decay) of an intervention’s impacts are not frequently undertaken [10,11,16,18].

Although many recommendations for and results of effective obesity preventive programmes have been described and published [7,8,10,11,14,15,16,18,19,20,21], the worldwide increase of obesity in children continues. This fact has amplified the interest in implementing longitudinal obesity preventive programmes (≥1 yearlong), with at least one follow-up (after ≥1 year) repeated measurement of obesity incidence [10,11,17,19]. However, published reports of such longitudinal studies seem to be very scarce globally [22,23].

Two follow-up studies of obesity prevention programmes undertaken in New Zealand [22] and in Germany [23] linked the duration (at least 1-year long) of a PA-enhancing and healthy nutrition intervention with at least one measurement of obesity incidence ≥2 years after cessation of the intervention. A follow-up analysis of the 2-year community-based obesity A Pilot Programme for Lifestyle and Exercise (APPLE) prevention project in New Zealand found that intervention children had considerably lower BMI z-scores and were less likely to be overweight than control children, but only for participants who completed the full-length 2-year intervention [22]. In Germany, a 4-year follow-up outcome evaluation of the school-based Kiel Obesity Prevention Study (KOPS) project found sustainable positive effects on nutritional knowledge, along with a significant remission effect of being overweight/obese in 172 intervention girls aged 10 years [23].

Whilst longitudinal obesity preventive studies (≥1-year long) with at least one follow-up (after ≥1-year) measurement in school-aged children have been implemented in Western countries [23], there is a notable lack of such studies across the Central and Eastern European nations (e.g., in the Czech Republic) where such research seems non-existent. The present study follows up on a previously published paper that describes the effects of a 2-year-long school-based PA intervention programme on the reduction of incidence of overweight and obesity in 176 Czech children aged 6–9 years [8]. Before the start of the two-year PA intervention (in 2006), there were no differences in the intervention and control groups. At the end of the PA intervention programme (in 2008), there was no occurrence of obesity in the intervention group, whereas more than one in five children in the control group was obese [8]. The present paper bridges the research gap of insufficient information in Central European nations of the effects of a longitudinal (≥2-year long) and objectively monitored ambulatory school-based PA programme on childhood obesity, with a two-year time lag after completion of the controlled PA intervention.

The aim of this study is to assess the effect of a two-year (2006–2008) non-randomised school-based PA intervention programme on reducing overweight and obesity two years after the end of controlled intervention (2010). The specific objectives are to: (i) describe the school days’ step counts during the school time and leisure time of the intervention and control children; (ii) examine the prevalence of overweight and obesity in the intervention and control children; (iii) determine the effect of children’s participation in the school-based PA intervention programme on the odds of obesity and overweight at a follow-up examination two years after the programme’s cessation.

## 2. Methods

### 2.1. Study Design

The Ethical Committee of the Faculty of Physical Culture, Palacky University in Olomouc approved the study. The children’s parents, their class teachers and management representatives of schools that had participated in the initial study [8] were informed of the objectives of the follow-up research. Free and voluntary (no financial incentives were provided) participation of children in the research was documented by a written approval form signed by their parents. The design of the initial study (a two-year non-randomised longitudinal school-based PA intervention programme) has been previously detailed [8]. The current study included the children who completed the entire two-year longitudinal non-randomised school-based PA intervention programme and who also participated in the follow-up examination two years after the end of the intervention programme (Table 1).

**Table 1:** School term dates of PA monitoring, numbers and BMI (M ± SD) of participants during the years 2006–2010.

		Baseline	Controlled PA intervention		Follow-up	
		Grade of primary school (calendar year)				
		1st (2006)	2nd (2007)	3rd (2008)		5th (2010)
Term Dates (day.month)		5.9–26.9	10.9–26.9	4.9–25.9		13.9–24.9
Number (BMI kg/m^−2^)						
Intervention	Girls	43 (15.3 ± 2.3)	43 (15.8 ± 2.2)	43 (16.2 ± 2.0)		43 (17.4 ± 2.1)
	Boys	45 (15.4 ± 1.9)	45 (15.7 ± 1.7)	45 (16.1 ± 1.6)		45 (16.9 ± 1.8)
						
Control	Girls	41 (15.4 ± 2.2)	41 (16.1 ± 2.4)	41 (17.0 ± 2.6)		41 (18.1 ± 2.5)
	Boys	47 (15.4 ± 1.5)	47 (16.1 ± 1.7)	47 (16.9 ± 1.9)		47 (17.9 ± 2.0)

PA—physical activity; BMI – Body Mass Index; M—mean; SD—standard deviation.

### 2.2. Sample

The selection of two primary schools for the school intervention PA was controlled because the curriculum at these two schools supported PA within a ‘School for Health’ project, entitled ‘Healthy Schools’ [24]. This ‘Healthy Schools’ project was developed by the World Health Organization for Europe (the Czech Republic Ministry of Education, Youth and Sports adopted the project in 1991) in response to the increased unhealthy behaviours among school-aged children. The ‘Healthy Schools’ project included many activities/strands (e.g., healthy diet, drug prevention, sexual risk behaviour, sports and singing competitions, poetry reading contests, school trips, and PA programmes). The two selected intervention schools met the same four non-PA criteria (drug prevention, sexual risk behaviour, singing competitions, poetry reading contests) of the ‘Healthy Schools’ project. 18% of Czech primary schools are involved in the ‘Healthy Schools’ project [24]. Table 2 lists the environment and equipment of the interventions and control schools. Control schools with PA unfriendly environments are representative of other schools in the area and present a suitable comparator.

The participation of students of the intervention and control primary schools in anthropometric and PA measurements was a regular part of the school curriculum. Upon completion of the one-week PA monitoring, each participating student received individual feedback on the results. Apart from tables and figures, recommendations for further health promoting PA were provided. The results were explained in the subjects of ‘Health Education’ and ‘Basics of Humanities and Natural Science’. Aggregate anonymized results were also presented to the parents of the participating children in parent meetings. In total, 88 children of the intervention group (43 girls and 45 boys) and 88 children of the control group (41 girls and 47 boys) were eligible for assessment of the effect of a two-year (2006–2008) non-randomised school-based PA intervention programme on reducing overweight and obesity two years after the end of controlled intervention (2010). Calendar age was equivalent between the intervention and control groups of children and no children were from an ethnic minority.

**Table 2:** PA Environment and school day PA content of intervention and control schools during two years (2008–2010).

		Description and Examples	
		Intervention Schools	Control Schools
		PA Environment	
		Involved	Not Involved
PA-friendliness		Environment/equipment more PA-friendly compared with control schools	Environment/equipment less PA-friendly compared with intervention schools
Environment/Equipment		Larger pitch with artificial turf	Smaller concrete pitch
		Playground with safe climbing frames, swings and a sandpit	Small playground without age adapted equipment
		Grassy area and school yard separated from public areas	No grassy areas
		Larger gymnasium with various PA aids and equipment	Smaller gymnasium without age adapted aids and equipment
		PA corners in corridors and classrooms or rooms designed for board and table games	Standard corridors without PA corners or classrooms designed for games
**Type (duration)**	**Frequency**	**PA Content**	
PE lessons(45 min)	2 per week	Overall development of children’s motor activity and fitness though movement games, simplified sports games, gymnastic and athletic exercises, and constitutional in co-educational teaching	
Short school break(4 min)	1 per day	Breathing and stretching exercises in classroom	Painting, drawing, writing in classroom
Long school break(20 min)	4–5 per week	Movement playing in corridors/room for table and board games	Painting, drawing, writing in classroom
After-school care(40-90 min)	each day	Movement and simplified sports games, exercises with PA aids and equipment in playground/gym	Painting, drawing, singing, doing homework, reading, playing board games in classroom

PE—physical education; PA—physical activity.

### 2.3. School-Based PA Programmes during Controlled Intervention and Follow-up

The baseline anthropometric and PA measurements were taken shortly after the children entered the first grade of intervention and control schools (September 2006), prior to the start of school-based PA programmes. The numbers and BMI of intervention and control children along with the dates of the annual monitoring of 7-day PA are presented in Table 1. Upon completion of the first baseline measurements, the control group children continued the standard school PA programme, whereas the intervention group children started the intervention PA programme (October 2006).

Table 2 lists the PA content at the control and intervention schools on school days during the two years (2008–2010) after the end of the intervention (2006–2008). The control schools implemented a standard school PA programme during the two years of the intervention (2006–2008) that comprised two mandatory 45-minute Physical Education (PE) lessons per week undertaken in the gym/playground. The standard school PA programme also had other potential PA opportunities (e.g., during recess periods and after-school care); however, the control schools seldom used such opportunities. Both control schools continued with the same school PA programme design after the end of the intervention (2008–2010).

In regards to the intervention schools, with the exception of two mandatory 45-minute PE lessons, the core PA within the school PA intervention programme was particularly during the school breaks and during the after-school care that immediately followed the end of a free lunch hour. In both intervention schools, during both the longer school breaks (20 min, 4–5 per week) and the after-school care (40–90 min daily), both individually and in small groups, children were offered various PA aids and equipment (skipping ropes, hoops, foam balls, soft balls, volleyballs, soft tennis and badminton rackets, age-adapted baseball bats and balls, hopscotch, ropes, flying discs, kick scooters) to be used in a gym or in the larger pitch or playground [8]. During the after-school care, playing with PA aids and equipment was enriched with physical games (e.g., tag, PA rhymes, hide-and-seek, and dodge ball) and age-adapted sports games (throw ball, football and floor ball played on a smaller pitch with smaller goals). During these games, children were allowed to freely choose not only the content but also the intensity of the selected PA according to their preferences, fitness, agreement with teammates and current weather conditions or available sports aids and equipment. In both intervention schools, the school PA programme was implemented in a friendly and creative atmosphere between the school management members, teachers and the researchers and research assistants (students on teaching practice). At the end of the 2-year intervention, both intervention schools accepted, adapted and embedded the design of the PA intervention programme within the regular daily curriculum.

### 2.4. Physical Activity Monitoring and Determining Overweight and Obesity

The 7-day PA monitoring, measuring and determining of main anthropometric indicators (body weight, body height, BMI, obesity, overweight, calendar age) of all participating children were performed repeatedly during September of 2006, 2007, 2008, and 2010 (Table 1). PA was monitored using a Yamax Digiwalker SW-200 pedometer (Yamax Corporation, Tokyo, Japan) and an individual PA log book [24] for at least twelve continuous hours a day during seven consecutive days. The PA log book was validated against the daily duration of PA from the Actigraph accelerometer in 7–11 year-old children (r_S_ = 0.81, *p* < 0.001) [24].

The Yamax Digiwalker SW-200 is a commercially available, small and light (1.5 × 3.5 × 5.0 cm; 20 g) electronic pedometer for vertical oscillation measurement. Its principle is the ON/OFF switching of an electric circuit using a spring-loaded pendulum arm moving during vertical oscillations resulting from a walking movement [25,26]. Each vertical oscillation exceeding the device threshold (#0.35g) is counted as a step. Overall step counts (the most accurate variable representing PA from the pedometer [26]) are shown on the device display. A variable school day step count comprises the sum of the school time step counts and leisure time step counts. A school time step count indicates the amount of steps at school (daily curriculum and after-school care). A leisure time step count represents the sum of the amount of steps conducted before the start of school and the amount of steps from departure from school to the evening. The Yamax Digiwalker SW-200 provides a reasonable assessment of a child’s daylong PA [27,28], PA during a certain part of the school day [29] and during walking, running, and physical games (tag, hopscotch) [30]. The Yamax Digiwalker SW-200 was validated against energy expenditure from oxygen consumption VO_2_ in 8-11-year-old children (r_S_ = 0.78–0.92, *p* < 0.001) [30]. However, pedometers are used only when the total amount of PA is of interest [31].

The anthropometric indicators were measured two to seven days prior to PA monitoring to prepare the individual PA log book for each participant. Calendar age was calculated from date of birth until the first monitoring day. Body height of children was measured using an A-319 Anthropometer (Trystom, Olomouc, Czech Republic). Body weight was measured using Tanita WB 110 S MA calibrated scales (Quick Medical Corporation, Seattle, WA, USA). Body height and weight measurements were performed in the morning during the first lesson at primary school with measurement accuracy to the nearest 0.5 cm and 0.1 kg. BMI was calculated as body weight (kg) divided by the square of body height [m]. Obesity, overweight and normal body mass were classified using percentile BMI charts for girls and boys aged 5 to 19 [32], where overweight and obesity represented the 85–97 and >97 percentiles, respectively, of age-differentiated BMI.

The procedure of PA monitoring (Yamax pedometer and personalised individual PA log book) was the same as in the previous study that assessed the effectiveness of a non-randomised school-based 2-year PA intervention in reducing obesity and overweight in 6- to 9-year-old children [8]. On the first day of the 7 days of PA monitoring, each child was provided with an elasticised belt with a pocket for the Yamax pedometer, a pencil and a personalised individual PA log book. The Yamax pedometer was not reset throughout the day. The personalised individual PA log book included the chronological structure of the day according to the current school schedule to record the time and value shown on the display (step count) of the Yamax pedometer. Children were instructed to wear the belt with the pedometer all day, for at least twelve hours a day, except during rest time, sleep, personal hygiene and bathing [24]. The research team instructed the students of Palacky University on teaching practice and trained the teachers and parents how to check, during the monitoring periods, the correct pedometer attachment and the correct reading and recording of the pedometer display data into the individual PA log book.

### 2.5. Statistical Data Processing and Interpretation

The data were analysed using SPSS v19.0 software (IBM SPSS, Inc., Chicago, IL, USA) and STATISTICA v.9 (StatSoft, Prague, Czech Republic). Group-related data were analysed in aggregate for both intervention schools (and in aggregate for the control schools) as the TwoStep cluster analysis and decision tree analysis did not show any indicators for clustering by individual intervention schools (individual control schools) during the PA programme (2008) and follow-up (2010) measurement. Two two-way (group × genders) analyses of variance (ANOVA) for repeated measures were conducted to examine the PA programme (2008) and follow-up (2010), and gender effects on step counts. School and leisure time PA of working days were used as dependent variables to thoroughly examine the PA programme and follow-up, and the effects of gender on step counts. To identify the differences in step count between control and intervention children at the different times of day (school × leisure time), Tukey’s HSD *post-hoc* test was used. The chi-square test compared the prevalence of obesity between the intervention and control groups in the follow-up measurement. Logistic regression (Enter method) was used to determine the obesity and overweight occurrence prospect over the course of implementation of the PA intervention and follow-up. The tested model included independent variables, such as affiliation with a group (intervention* vs.* control) and gender (girls* vs.* boys). *P*-values < 0.05 were considered statistically significant. The strength of the relationship between the independent (affiliation with a group, gender) and the dependent variable (step counts) in school and leisure time of working days was assessed by means of “effect size” *d* coefficient for repetitive measures [33], where values *d* = 0.2, 0.5 and 0.8 may be interpreted as a small, medium and large effects, respectively [34].

## 3. Results

**Figure 1:**
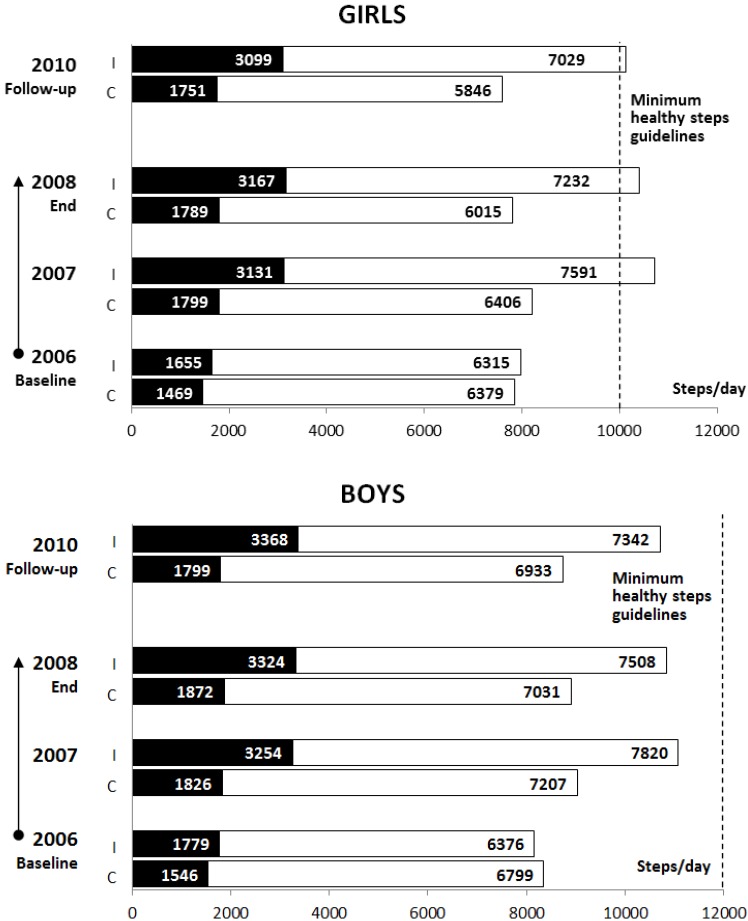
Mean school day step counts of intervention and control children throughout the intervention and follow-up (■—school time; □—leisure time; I—intervention; C—control).

According to the ANOVAs for repeated measures, a significant positive effect of the intervention PA programme was found two years after the completion of the controlled intervention for school time steps and leisure time steps for intervention children (*F*(1, 176)_SCHOOLtime_ = 83.26, *p* < 0.0001, *d* = 1.38; *F* (1, 176)_LEISUREtime_ = 12.75, *p* < 0.0005, *d* = 0.52) when compared with the control children (Figure 1). Gender had a significant effect on the leisure time steps (*F* (1, 176)_LEISUREtime_ = 4.53, *p* = 0.0348, *d*_G_ = 0.80, *d*_B_ = 0.34). Tukey’s HSD post-hoc test indicated that both intervention girls and boys achieved higher school time step counts (*p*_G_ < 0.0001, *d*_G_=1.45; *p*_B_ < 0.0001, *d*_B_ = 1.33) than control girls and boys. With a 2-year time lag after completion of the intervention PA programme, the Tukey’s HSD *post-hoc* test revealed a significant decrease in leisure time steps counts in the intervention children (*p*_G_ < 0.0001, *d*_G_ = 0.80; *p*_B_ < 0.0001, *d*_B_ = 0.22). Nevertheless, their leisure time steps were significantly higher (*F* (1, 176)_GROUP_ = 12.75, *p* < 0.0006, *d* = 0.52) compared with the controls (Figure 1). At follow-up measurement in comparison with the end of the controlled intervention, there was only a slight decrease in the proportion of intervention children (girls: 55.8%* vs.* 51.2%, and boys: 26.6%* vs.* 22.2%) who achieved the minimum national Czech PA guideline for maintaining health for children aged 10–12 years (steps per day: 10,000 girls and 12,000 boys) (Figure 1) [24]. For controls, there was a progressive decrease in the proportion of children who achieved this minimum of national PA guidelines (girls: 4.8%* vs.* 2.4%, and boys: 6.4%* vs.* 4.3%).

**Figure 2:**
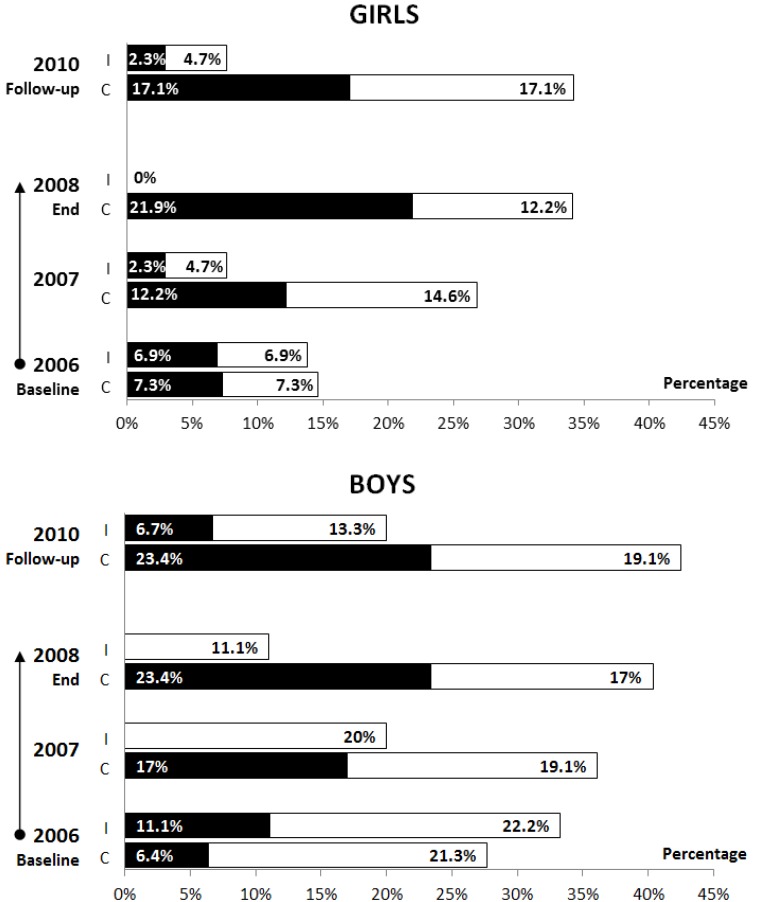
Mean percentages of obese and overweight intervention and control children throughout the intervention and follow-up (■—obese; □—overweight; I—intervention; C—control).

Even two years after the completion of the controlled PA intervention programme, obesity prevalence in the intervention group (2.3%_G_ and 6.7%_B_) assessed by the chi-square test was significantly lower (*p* < 0.05) compared with the control group (17.1%_G_ and 23.4%_B_) (Figure 2). The prevalence of overweight and obesity in a representative sample of Czech 11-year-old schoolchildren in 2010 was 15.7% for girls and 30.7% for boys [4]. While the prevalence of overweight and obesity in children from the intervention group was lower than the prevalence of overweight and obesity in a representative sample of Czech 11-year-old schoolchildren, the prevalence of overweight and obesity in the control group exceeded the Czech national reference data of overweight and obesity in schoolchildren.

A significant (*p* < 0.001) impact of children’s participation in the school-based PA intervention programme on the odds of decreasing obesity and overweight prevalence was found two years after the end of the controlled PA intervention programme, in autumn 2010 (Table 3). In the follow-up measurement, it was recorded that the odds of overweight and obesity in the intervention children was four times lower than the controls.

**Table 3:** Impact of participation in intervention programme on the odds of child obesity/overweight during 2006–2010.

		Baseline		Controlled PA intervention				Follow-up	
		Grade of primary school (calendar year)							
		1st (2006)		2nd (2007)		3rd (2008)		5th (2010)	
Group	N	OR	CI	OR	CI	OR	CI	OR	CI
Control	88	Ref.		Ref.		Ref.		Ref.	
Intervention	88	1.17	0.57–2.40	0.34 *	0.16–0.72	0.09 ^‡^	0.04–0.27	0.25 ^‡^	0.12–0.53
Gender									
Girls	84	Ref.		Ref.		Ref.		Ref.	
Boys	92	2.64 *	1.24–5.62	1.99	0.94–4.20	1.85	0.83–4.12	1.85	0.90–3.80
R^2^		0.06		0.10		0.25		0.14	

N – number of participants; OR – odds ratio; CI – confidence interval; Ref. – reference group; statistical significance * *p* < 0.005, ^‡ ^*p* < 0.001; R^2^ – Nagelkerke coefficient of determination, logistic regression model, Enter method.

## 4. Discussion

The current study assessed the effectiveness of a longitudinal (2006–2008) non-randomised school-based PA intervention programme on childhood obesity in children aged 10–12 years two years after its completion (2010). Hence, this study bridges the gap of the sparsely available longitudinal studies in Central and Eastern European nations aimed at decreasing obesity in younger school-aged children using increased levels of objectively monitored ambulatory PA in a school environment followed by an analysis of obesity prevalence after an appropriate time lag following completion of the controlled PA intervention.

In relation to the first objective of the current study, in the follow-up measurement, intervention children achieved on average more than 2,200 steps per day than controls; however, even with such accomplishments, only the intervention girls achieved the minimum of the national PA guidelines for maintaining health for Czech children aged 10–12 years (10,000_G_ and 12,000_B_) [24]. From the point of increased PA and decreased % of overweight/obesity in follow-up measurement, the intervention girls achieved better results than the intervention boys. This finding is supported by the main conclusions of a systematic review of moderators of 61 school-based interventions [35], which claims that female gender is one of the most positive moderators of the intervention effects. Unfortunately, the average school day step counts of control and intervention children at follow-up (September 2010) was lower when compared with step counts of 6- to 12-year-old girls (10,800–14,800) and boys (11,500–18,100) from Australia, Canada, the Netherlands, New Zealand, Great Britain, Japan and the United States of America [36,37]. The lower daily step counts in our sample could also have been influenced by the fact that our sample, as opposed to children from the other countries mentioned, wore the Yamax pedometer in a pocket of an elasticized belt, which could have had an effect on the sensitivity of the device. The children of other studies [36,37] wore the pedometers tightly bound to the body and did not use special pockets.

In terms of school time step counts, at the 2-year follow-up the measurements reached 30.8% of overall school day PA in the intervention children and 21.9% in the controls. The difference of ≥8% in the proportion of school PA in overall school day PA between intervention and control children might be due to the regular daily curriculum of both intervention schools which continued on with an adapted design/version of the initial PA intervention programme during 2008–2010, after the end of the initial intervention (Table 2). For instance, the teachers of the intervention schools acquired all types and variants of movement games, simplified sports games, and exercises, and they also created other games and used new teaching movement aids. According to the opinions of the teachers of the intervention schools, the follow-up participation in the school PA programme for the intervention children was both a voluntary and joyful activity. Contrary to the results of a 5-year longitudinal study of Australian children aged 5–12 years [38], the present 2-year follow-up study found no significant decreases in recess and lunchtime PA in children aged 7–12 years in the intervention or control groups. However, the core of the follow-up school time PA was primarily in after-school activities. Moreover, Ridgers, Timperio, Crawford and Salmon [38] monitored school PA of children with an uni-axial ActiGraph accelerometer, which provides more accurate detection of intensity of PA than the Yamax pedometer employed in the current study [39,40,41]. Similar to the findings of studies of Belgian children aged 10–12 years [42] and of English aged 6–12 years [43], it could be argued that providing game equipment in PA-friendly environment (playground, gym and corridors) is an effective strategy to increasing children’s PA levels at primary schools. In relation to results of a longitudinal 10-year PA and nutrition intervention program in Crete [44], we are inclined to agree with findings that 2-year participation of increased school PA positively affected subsequent PA after cessation of controlled non-randomised school-based PA intervention programmes.

As for objective two, we compared the prevalence of overweight and obesity in the intervention and control children 2-years after the end of the non-randomised school-based PA intervention programme. It needs to be noted that during the two years of the non-randomised school-based PA intervention programme, obesity in the intervention group children was eliminated, and the same was true of overweight in girls (Figure 2). On the contrary, the proportion of control children with overweight and obesity continuously increased, in line with Czech national trends [4] and also American trends [2].

Our positive findings as regards to obesity reduction in the intervention children in the follow-up measurement are in agreement with the results of the longer 2-year APPLE project in New Zealand [22]. The APPLE project represented a multifaceted intervention, aimed at increasing PA and the intake of fruits and vegetables, whilst reducing the consumption of sugary drinks [22]. Only among the intervention children aged 10–12 years who participated in the full length of the 2-year APPLE intervention were considerably lower BMI z-scores and lower ratio of overweight compared with controls during the follow-up measurement 2 years after the end of programme [22].

In connection with objective three, the current study assessed the effect of participation in a non-randomised school-based PA intervention programme on the odds of obesity and overweight prevalence at a follow-up examination two years after the end of the intervention. Presented findings indicated that intervention children were distinctly less likely and four times less likely to be obese/overweight than control children at the follow-up measurement. The results for the odds of being obese/overweight at follow-up measurement (2010) were slightly more positive than the findings of the APPLE prevention initiative [22]. We conclude that these more positive findings were most likely because the teachers of both of the schools that hosted the school-based PA intervention programme adopted it and continued on with it without the direct involvement of the research team. Although the intervention programmes combining increased PA and a suitable diet seem to be more effective in childhood obesity reduction [20,21], the presented results suggested that repeated 4-year-long preferred PA performed in a safe school environment provided with suitable PA equipment allows and contributes to a significant decrease of childhood obesity. A helpful approach of school teachers, a PA-conducive and friendly school environment [9,42,43], and 4-year-long repeated realisation of everyday school PA were the preconditions for low odds of obesity/overweight prevalence in children including the two years following the completion of direct involvement of the research team in the school-based PA intervention programme.

## 5. Limitations and Future Research

The current study has limitations. The study design (non-randomised controlled trial) and the small sample size of intervention and control groups of children require cautious generalisation with respect to extrapolation of these results to the wider population of children in the Czech Republic. Determining the level of body weight using an age-differentiated BMI percentile chart without more accurate knowledge of body composition or current biological age of the children might complicate data interpretation for borderline individuals. A further limitation is that this study did not monitor the eating habits of the children, as changes in eating habits might have influenced obesity levels. A potential measurement bias could have been caused by the fact that data collectors were not blinded to the study outcomes. Other descriptive characteristics of the intervention and control children and their families (socio-economic status, level of body weight of parents) would have also been helpful for more accurate assessment and explanation of the effectiveness of a school-based PA intervention programme and 2-year follow-up measurement on reducing overweight and obesity of 10 to 12-year-old Czech children. However, despite these limitations, the repeated objective monitoring of long-term PA in a school context supports the conclusions of the study.

Future research should analyse long-term follow-up effects of multi-component school-based and family-involved interventions [45,46] on reducing childhood obesity employing more accurate and reliable multi-functional PA monitoring tools, e.g., ActiGraph wGT3X+ or ActiTrainer monitor [47,48] to uncover the intensity and length of performed PA. Critical meta-analysis of such evidence-based follow-up studies could provide answers to the question of the optimal length and intensity of PA to effectively reduce obesity in children long-term.

## 6. Conclusions

The current study indicates favourable effects of the daily school-based PA intervention programme on lower prevalence of overweight/obesity, which was maintained two years after the end of direct involvement of the researchers. Two years of direct involvement of the researchers in the school-based PA intervention programme seems to be sufficient time to adopt the design and content of intervention programme by the intervention schools’ staff members.
